# Evaluating human versus machine learning performance in classifying research abstracts

**DOI:** 10.1007/s11192-020-03614-2

**Published:** 2020-07-18

**Authors:** Yeow Chong Goh, Xin Qing Cai, Walter Theseira, Giovanni Ko, Khiam Aik Khor

**Affiliations:** 1 School of Mechanical and Aerospace Engineering, Nanyang Technological University, Singapore, Singapore; 2 School of Business, Singapore University of Social Sciences, Singapore, Singapore; 3 School of Economics, Singapore Management University, Singapore, Singapore; 4 Talent Recruitment and Career Support (TRACS) Office and Bibliometrics Analysis, Nanyang Technological University, Singapore, Singapore

**Keywords:** Discipline classification, Text classification, Supervised classification

## Abstract

We study whether humans or machine learning (ML) classification models are better at classifying scientific research abstracts according to a fixed set of discipline groups. We recruit both undergraduate and postgraduate assistants for this task in separate stages, and compare their performance against the support vectors machine ML algorithm at classifying European Research Council Starting Grant project abstracts to their actual evaluation panels, which are organised by discipline groups. On average, ML is more accurate than human classifiers, across a variety of training and test datasets, and across evaluation panels. ML classifiers trained on different training sets are also more reliable than human classifiers, meaning that different ML classifiers are more consistent in assigning the same classifications to any given abstract, compared to different human classifiers. While the top five percentile of human classifiers can outperform ML in limited cases, selection and training of such classifiers is likely costly and difficult compared to training ML models. Our results suggest ML models are a cost effective and highly accurate method for addressing problems in comparative bibliometric analysis, such as harmonising the discipline classifications of research from different funding agencies or countries.

## Introduction

The classification of science is a fundamental research question in scientometrics (De Bruin and Moed 1993). Proper evaluation of the quantity and impact of scientific output must take into account differences in the distribution of citations across disciplines (Radicchi et al. 2008). This is a central concern when measuring the performance of individual researchers (Piro et al. 2013), journals (Moed 2010), universities (Moed 2006), funding bodies (Robitaille et al. 2015), or even countries (King 2004). Moreover, when comparing performance across units of evaluation such as universities, funding agencies or countries, differences in disciplinary profiles between them require mapping research output onto a common classification.

The most straightforward method for classifying research publications into disciplines is to have researchers or assistants do so but this is very time-consuming and manpower-intensive. Recent advances in natural language processing (NLP) machine learning (ML) techniques (Wei and Croft 2006; Aggarwal and Zhai 2012) have made automatic classification of disciplines possible (Yau et al. 2014; Freyman et al. 2016) but no study to date has analysed the relative performance of ML versus human classification of research into *pre*-*existing schemes*. Classification into existing discipline schemes applies to contexts where scientific research is evaluated and administered according to a framework or agenda specified in advance by policymakers or administrators. For example, in the context of the current COVID-19 virus pandemic, policymakers might want to evaluate the strength of a country’s research capabilities in specific biomedical areas relating to virology and immunology.

Our study shows that ML classification algorithms trained on an existing mapping of abstracts onto fixed discipline groups outperform human research assistants at classifying new abstracts into these discipline groups. The accuracy, measured by F1 score, of ML classifiers is 2–15 standard errors higher than that of human classifiers, with reliability, as measured by Fleiss’ *κ,* also being consistently higher for ML than for humans.

## Literature review

One of the central questions in the bibliometric measurement of the quantity and impact of research output is how to properly account for differences across disciplines. The real-world implications of this question range from career outcomes and funding allocation to national and supranational research policy. For example, individual researchers’ tenure and promotion evaluations should take into account differences in the average number of publications and citations per researcher across disciplines (Piro et al. 2013), while rankings of universities (Moed 2006) and comparisons of funding agencies (Robitaille et al. 2015) should take into account differences in disciplinary profiles and priorities.

The classification of science is also referred to as discipline clustering and discipline mapping in the literature, both referring to the use of statistical algorithms to automatically classify research. The two approaches used in this literature are termed supervised and unsupervised classification. While both supervised and unsupervised classification are concerned with the automated classification of large corpora of texts based on a statistical analysis of the words used (Lee and Yang 2009), their methods and interpretation differ fundamentally. Supervised classification models are trained on a ground truth, which could be an existing classification carried out by subject-matter experts. The supervised ML classifier learns parameters based on the distribution of words in the ground truth that is used later to classify unclassified texts. Hence, accuracy has the natural interpretation of how well the classifier replicates the ground truth.

On the other hand, unsupervised classification, does not require any pre-existing ground truth. Instead, unsupervised classification depends on model parameters given by the researcher to determine how to score document similarity and compute cluster delineations. Because a natural ground truth does not exist, it has to be separately defined and justified by researchers to measure the accuracy of their unsupervised classifier.

Unsupervised classification has a longer history in bibliometrics. The earliest studies developed co-citation clustering (Small 1973) which uses the weighted network graph of citations to determine clusters of authors working in similar fields. This assumes that authors in similar fields cite similarly. In response to epistemic concerns (Oberski 1988), Callon et al. (1983) introduced co-word analysis. Co-word clustering calculates the semantic similarity of documents to determine clusters of papers in similar fields. This assumes that papers in similar fields use similar lexica to convey membership in a scientific community and efficiently transmit ideas. Co-word clustering also faces serious epistemic issues (King 1987).

Later, Braam et al. (1991a, b) use a hybrid of both methods to cluster 3400 publications in the *Chemical Abstracts* database and 1384 publications in the *BIOSIS* database. This line of research was further taken up in Liu et al. (2009, 2010, 2012), and others.

More recent studies use unsupervised *ML* algorithms. Examples are Yau et al. (2014) and Nichols (2014), who use latent Dirichlet allocation (LDA) to extract topic clusters. Freyman et al. (2016) used topic co-clustering to classify 277,818 US National Science Foundation (NSF) project abstracts. These studies update the discipline mapping literature by showing that well-studied unsupervised ML algorithms from the information retrieval literature also perform well for classifying scientific text, and benchmarking the classification performance of these algorithms.

The distinction between supervised and unsupervised classification is important because, for many policy applications, supervised classification is more relevant. For example, funding agencies such as the NSF and ERC require applications for funding to be mapped into their pre-existing disciplinary classifications. Such disciplinary classifications reflect policymakers’ and administrators’ funding priorities (including funding uniformity across disciplines) and administrative processes that are external to the research to be classified. The way such classification is currently applied is mostly manual, either by applicants or by subject-matter experts at the funding agencies or on evaluation panels (Herzog et al. 2016). Because of the time and manpower costs involved, funding agencies such as the NSF are investigating methods for automatic classification (Nichols 2014) but so far no study has directly compared the performance of ML algorithms to humans in terms of accuracy and reliability.

In other fields, studies that have compared human to automated supervised classification have found evidence in favour of automated classification methods. These studies are in fields as diverse as haematology (Simundic et al. 2009), software engineering (Schumacher et al. 2010), and online opinions mining (Weismayer et al. 2018). Automated classification performance is generally shown to be at least as accurate as human classification, and significantly more reliable based on inter-rater reliability (IRR) analysis. Nevertheless, it is not clear that supervised ML classification methods can be accurately applied beyond the domains previously studied, such as medical specimen assessment and opinions mining, to scientific abstracts. Whether ML performs as well at classification of textual data from scientific abstracts into pre-existing schemes is therefore an open question, and our study fills this gap.

## Methodology and data

### Research questions

We designed a study to answer three distinct but related questions: (1) Is ML more *accurate* than humans at classifying scientific abstracts? (2) Is ML more *reliable* than humans at classifying scientific abstracts? (3) To what extent does human classification performance improve, relative to ML, through (i) increased task training, (ii) increased prior knowledge, (iii) selection on past performance, and (iv) feedback? In this study, accuracy means “classifying an abstract correctly to its true discipline group”, while reliability means “two classifiers classifying an abstract to the same discipline group, whether or not this classification is correct”. Accuracy and reliability are not necessarily related, although as any group of classifiers approach perfect accuracy then trivially they also approach perfect reliability.

### Data

We use the abstracts of European Research Council (ERC) Starting Grant (StG) funded projects that were accepted between 2009 and 2016 inclusive. The ERC evaluation panel structure has been stable since 2008 (European Research Council 2019a). For the purpose of our study, the existing panel classifications are considered the ground truth that we do not question. ERC grant applicants initially select the panel they apply to. While panel chairs may re-assign an application to a different panel, and may consult members of other relevant panels, each application is nevertheless assigned to a single panel (European Research Council 2019b).

As we are primarily interested in the classification of natural sciences abstracts, we focus on the 19 physical and life sciences panels, comprising 2523 abstracts in total.

Table 1 presents these panel codes and titles.

**Table 1:** Codes and titles of ERC evaluation panels

Code	Title	Code	Title
PE1	Mathematics	LS1	Molecular Biology, Biochemistry, Structural Biology and Molecular Biophysics
PE2	Fundamental Constituents of Matter	LS2	Genetics, ‘Omics’, Bioinformatics and Systems Biology
PE3	Condensed Matter Physics	LS3	Cellular and Development Biology
PE4	Physical and Analytical Chemical Sciences	LS4	Physiology, Pathophysiology and Endocrinology
PE5	Synthetic Chemistry and Materials	LS5	Neuroscience and Neural Disorders
PE6	Computer Science and Informatics	LS6	Immunity and Infection
PE7	Systems and Communication Engineering	LS7	Applied Medical Technologies, Diagnostics, Therapies and Public Health
PE8	Products and Processes Engineering	LS8	Ecology, Evolution and Environmental Biology
PE9	Universe Sciences	LS9	Applied Life Sciences, Biotechnology, and Molecular and Biosystems Engineering
PE10	Earth System Science		

### Study design

Our study has four stages. In the Undergraduates stage, we recruited 63 undergraduate student assistants from Nanyang Technological University, a major research university in Singapore, for a full-day task. We sent out an email to recruit undergraduates for our research abstract classification study to all undergraduates via the university’s mailing lists. To screen potential classifiers for aptitude, we required applicants to complete a short example task to classify two research abstracts. Just over one hundred undergraduates responded to the email and completed the example task. While we made every effort to recruit each applicant, 63 eventually reported for work on the day of the study. The task was conducted at a classroom on campus, where they were given one of four training sets of abstracts in the morning. Each training abstract was labelled according to the existing ‘ground truth’ ERC evaluation panel, allowing assistants to study how the abstracts ought to be classified. In the afternoon, they were given a test set of different abstracts, with the ERC evaluation panel labels removed, and were told to assign each abstract to the ERC panel most likely to match the ‘ground truth’. To ensure that their performance is due to learning from their training set only, we disallowed peer discussion and internet use. To incentivise both performance and completion, they were compensated with a flat rate of Singapore $120 for a day of work plus a variable amount of $4 for every 10 abstracts in their test set that they correctly classify. In this stage, the average amount paid was $163, which was paid in cash.

In the second stage, termed the high-performance undergraduates stage, we retained eight undergraduates from each training set group with the highest accuracy scores. These undergraduates also had to be willing to continue with the study for up to two more stages. Over email, we gave these high-performance undergraduates another test set to classify without further training. This stage is meant to answer question (3)(iii) above about selection effects for human classification performance. After they returned the completed test sets, we began the third stage, termed the high-performance undergraduates plus feedback stage. We gave the high-performance undergraduates feedback on the actual ‘ground truth’ classifications of the abstracts they had classified in the previous two stages. Then we gave them a third test set to classify. This stage is meant to answer question (3)(iv) above on the effect of feedback on human classification performance. As before, we instructed the undergraduates to refrain from discussing or using the internet while classifying the test sets. The undergraduates were compensated with a flat rate of $150 for completing both test sets plus a variable amount of $6 for every 25 abstracts that they correctly classify. In this stage, the average amount paid was $207, which was paid in cash.

In the last postgraduates stage, we recruited 26 Ph.D. students and postdoctoral researchers in STEM disciplines (postgraduates) from Nanyang Technological University for a half-day task where they were given a test set to classify without any training or task exposure. We sent out an email on our recruitment of postgraduates for our research abstract classification study to all postgraduates in the College of Engineering through the assistance of the administrators in each School in the College. Nearly half of the Postgraduates were from the Engineering sciences. The task was conducted in a classroom on campus, and as with the undergraduates, discussion and internet use was not allowed. The postgraduates were compensated with a flat rate of $80 for completing the test set plus a variable amount of $10 for every 50 abstracts that they correctly classify up to a total of $120. The average amount paid was $98, and was paid in the form of $10 vouchers (rounded up) for a large national supermarket. This stage is meant to answer question (3)(ii) above on the effect of prior knowledge on human classification performance. Additional details on the study participants are in the “Appendix”.

### Test and training sets

Test and training sets are generated by stratified random sampling where an equal number of abstracts were sampled from each panel. From a pilot trial, we found that undergraduate classifiers were able to comfortably complete about two to three hundred abstracts in half a day. Hence, all our test sets consist of 247 abstracts, or 13 abstracts from each evaluation panel. In the undergraduates stage, all undergraduates were given the same test set to classify. In subsequent stages, we designed the test sets so that each human classifier would face a unique test set consisting of a common component of 95 abstracts (5 abstracts from each panel) and an individual, independently sampled component of 152 abstracts (8 abstracts from each panel). The common component addresses question (2) above about whether human or ML classifiers are more reliable. The individual, independently sampled component allows us to ensure the results are robust to idiosyncrasies in the common component abstracts. This is especially important for addressing question (1) on comparing ML accuracy to that of human classifiers, since any given ML model, once trained, will always produce the same classification output in response to the same test set. A variety of test sets is necessary to provide a more robust estimate of ML accuracy.

From the pilot trial we also found that undergraduate classifiers were able to comfortably study several hundred abstracts in half a day. Hence, to address question (3)(i) above about the effect of more training on human classification performance, in the Undergraduates stage we generated two large training sets with 380 abstracts, and two small training sets with 190 abstracts, and randomly assigned undergraduate classifiers to each training set. To generate the four training sets, we first created 20 randomly sampled sets of abstracts—10 small, and 10 large—and trained an ML classifier on each set. We then scored each trained ML classifier on the Undergraduates test set, and chose the four training sets that produced the best and worst performing ML classifiers, in the small and large training sets respectively. Table 2 summarizes the number of classifiers and the sizes of the training and test sets given in each stage.

**Table 2:** Summary of numbers of classifiers and sizes of training and test sets

Stage	Human classifiers^a^	Training set		Test set size	
		Code	Abstracts	Common	Individual
Undergraduates	16	A	380	247	0
	16	B	380		
	15	C	190		
	15	D	190		
High-performance undergraduates	7	A	380	95	152
	8	B	380		
	7	C	190		
	8	D	190		
High-performance undergraduates after feedback	7	A	380	95	152
	8	B	380		
	7	C	190		
	8	D	190		
Postgraduates	26	–	–	95	152

^a^Classifiers that are excluded during analysis are not counted (see “Data Exclusions”)

### ML classification

We use the support vector machines (SVM) algorithm as we found in Khor et al. (2018) that it has the best abstract classification performance among the basic supervised classification algorithms. The SVM algorithm finds the optimal hyperplanes that bisect the data to an “In” and “Out” classification for every category using a maximum-residual criterion (see Cortes and Vapnik 1995 for a detailed explanation). We combine SVM with bag-of-words pre-processing of the abstracts and use text frequency-inverse document frequency (TF-IDF) as our feature score (see Baeza-Yates and Ribeiro-Neto 1999 for a detailed discussion about information retrieval). For hyperparameter optimisation, we use grid search with cross-validation due to its ease of implementation. For an extended discussion of hyperparameter optimisation in machine learning, see Bergstra and Bengio (2012).

For a fair comparison of classification performance between human and ML classifiers for question (1) above, the training for our ML classifiers must be restricted to the same amount of training given to the human classifiers as reasonably as possible. In the undergraduate stages, we train an ML classifier using each of the four training sets. Undergraduate classifiers from each training set group are then compared only to the performance of the ML classifier that had been given the same training set. In the Postgraduates stage, no training sets are given to the postgraduate classifiers as their doctoral training is taken to be an extensive period of training in the knowledge and disciplinary boundaries of Science. To simulate extensive background training, ML classifiers in the Postgraduates stage for each test set are trained using all other abstracts that were left out of the test set (2276 abstracts in total).

### Measuring performance

While there are many measures of accuracy in classification problems, precision and recall are most often reported (Sokolova and Lapalme 2009). Precision is the ratio of true positives to the sum of true positives and false positives. Recall is the ratio of true positives to the sum of true positives and false negatives. Positive and negative refer to whether an abstract is classified into a given evaluation panel or not. Intuitively, precision says what proportion of our classifications are correct and recall says what proportion of the actual abstracts we classify correctly. Because both are important measures of accuracy, their harmonic mean, the F1 score, is our preferred accuracy metric. As precision, recall and F1 are defined only for a 2 × 2 confusion matrix, the overall precision, recall and F1 of a test set is the mean of the scores across all evaluation panels.

### Measuring reliability

The reliability of a group of classifiers is also known as inter-rater reliability (IRR), which measures to what extent different classifiers tend to classify the same abstracts to the same evaluation panel. Reliability does not measure whether classifications match the ground truth, only whether different classifiers agree on the same classification. Reliability is measured with Fleiss’ *κ* (1971) as our data contains more than 2 classifiers per test set. *κ* has an upper limit of 1, which represents perfect agreement, while 0 implies that the agreement rate is no better than pure chance. Negative values of *κ* imply disagreement beyond what would be expected by chance alone. For interpretation of *κ*, Landis and Koch (1977) proposed the following scale: *κ* > 0.4 is “Moderate” agreement, *κ* > 0.6 is “Substantial” agreement and *κ* > 0.8 is “Almost Perfect” agreement. For a detailed discussion of IRR, refer to McHugh (2012).

### Data exclusions

We exclude sets where the human classifier failed to complete at least 95% of the abstracts in their test set. 1 set in the undergraduates stage and 1 set in the high-performance undergraduates stage were excluded thus. We also excluded one human classifier who had 89% and 97% accuracy in the two High-Performance undergraduate stages. The extremely high performance of this classifier, both relative to their own prior performance and to that of other classifiers, suggested use of the internet (where all ERC abstracts and their evaluation panel assignments are searchable). This classifier’s data is retained in the first undergraduates stage, where there was no access to the internet possible.

## Results

### Overall performance in all stages

**Fig. 1:**
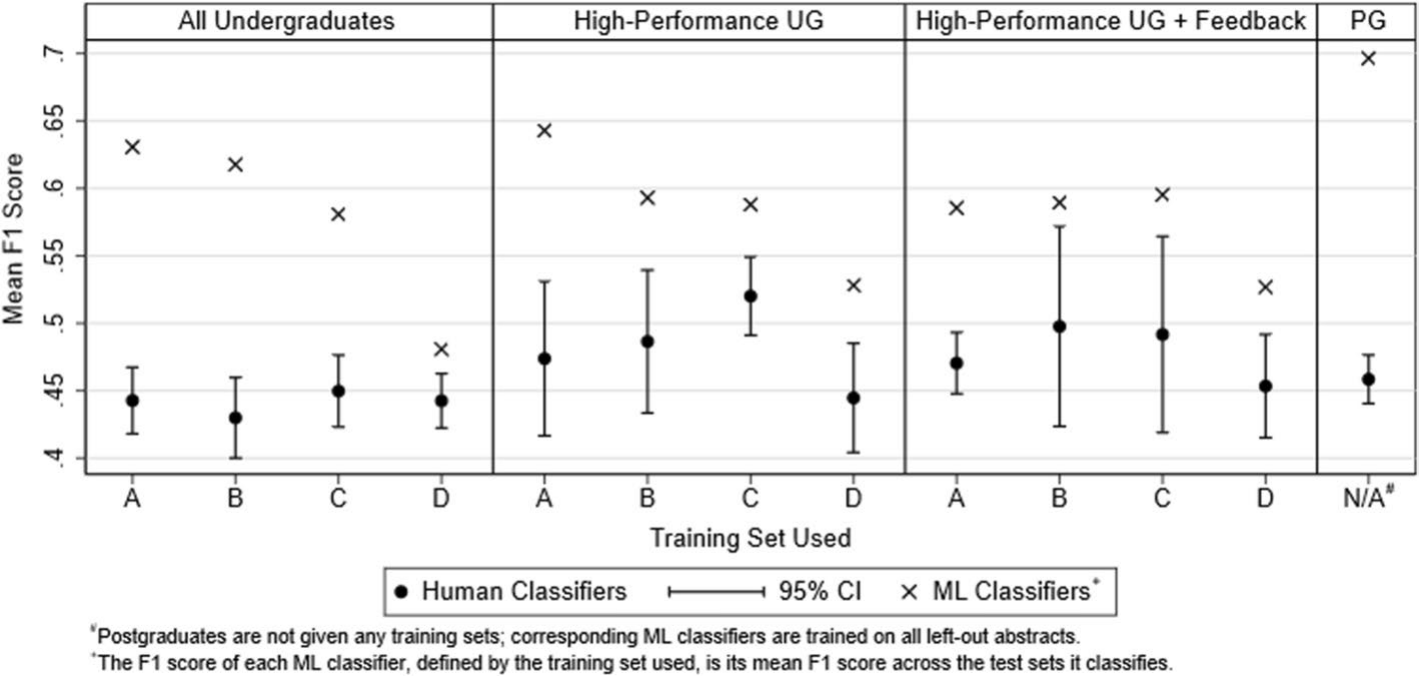
F1 scores for human and ML classifiers across each stage and training set

Figure 1 compares the average performance of human and ML classifiers, across each stage and by each training set. The 95% confidence intervals for the mean F1 score of human classifiers can be interpreted as two-sided *t* tests against the mean F1 score of the respective ML classifier at *α* = 0.05. ML classifiers perform significantly better than human classifiers at replicating the ground truth panel classifications across all stages and training sets. The ML classifiers are 2–15 standard errors better than undergraduate classification performance across all stages. In the Postgraduates stage, ML classifiers improves to 26 standard errors above postgraduate classification performance. This does not mean postgraduates perform worse than the average undergraduate in classification performance. Rather, this is driven by the increased performance of the ML classifiers in the postgraduates stage; recall that the ML training set in the postgraduates stage is the largest, consisting of all left-out abstracts (2276 abstracts), to attempt to match the postgraduate classifiers’ greater expertise.

### Performance of high-performance undergraduate and postgraduate classifiers

**Fig. 2:**
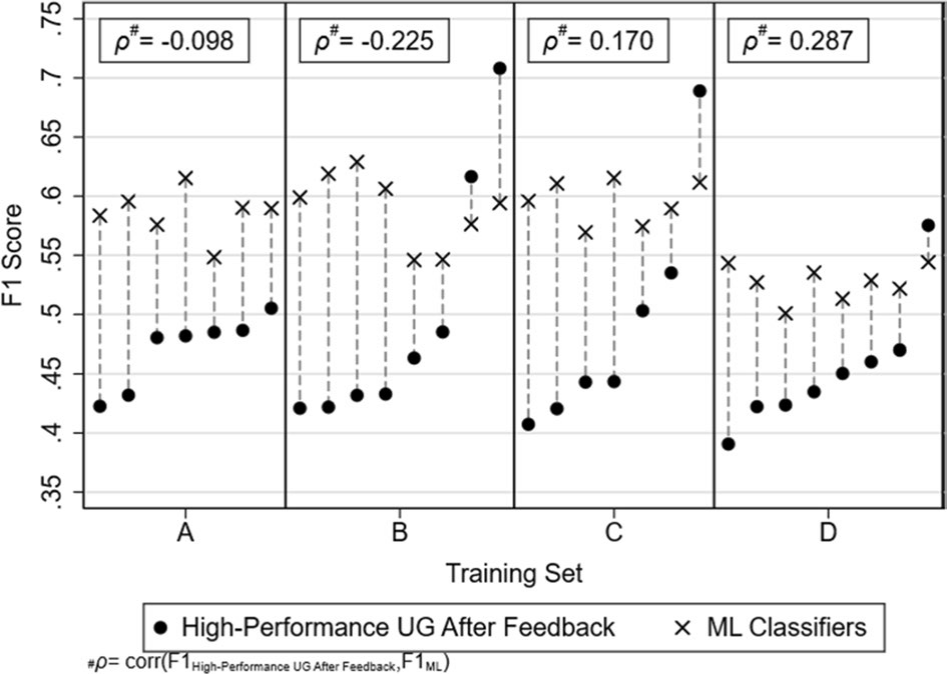
F1 scores of high-performance undergraduate classifiers after feedback and the corresponding ML classifiers

**Fig. 3:**
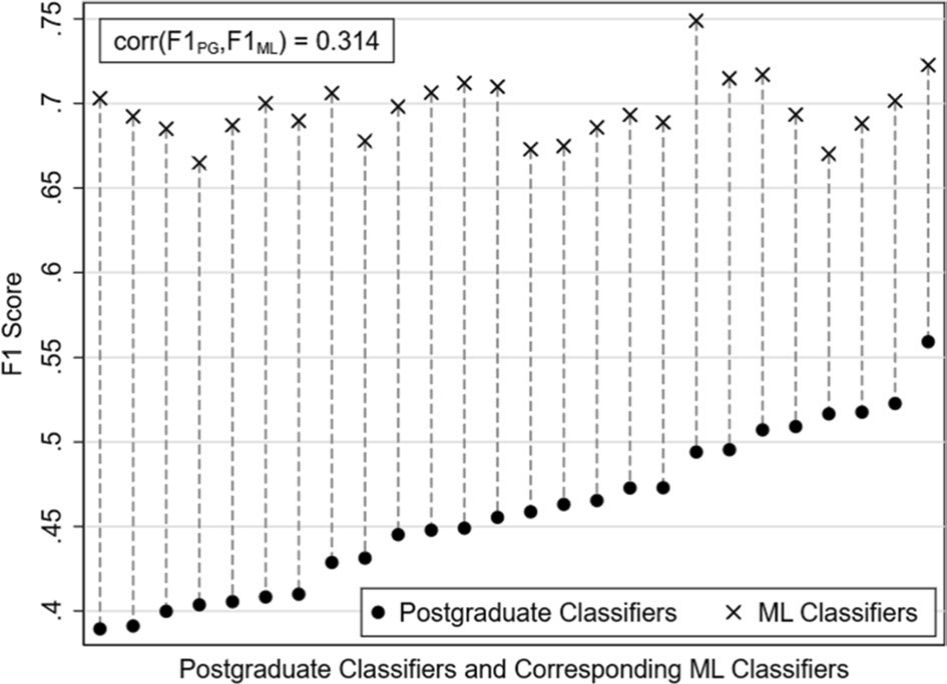
F1 scores of postgraduate classifiers and the corresponding ML classifiers

We turn to examining in detail high-performance undergraduate classifiers, after feedback in the third stage, in Fig. 2. Each undergraduate classifier is matched to an ML classifier that was trained on the same training set, and used to classify the same test set as themselves. Out of 30 high-performance undergraduate classifiers, 4 outperformed the ML classifier; 2 of these outperformed by at least 0.05 F1 score points—at least 5% points greater accuracy than the corresponding ML classifier. The performance of the top two undergraduates is similar to that of the performance of ML classifiers trained on the substantially larger training set of all left-out abstracts (see Fig. 3).

Figure 3 reports the performance of postgraduate classifiers, ranked by performance. Each postgraduate classifier is matched to an ML classifier used to classify the same test set. There is no matching on training sets, since postgraduate classifiers are assumed by design to already possess the requisite knowledge. None of the 26 postgraduates outperformed the ML classifier, although in general postgraduates had similar performance to that of the high-performance undergraduate classifiers, after feedback. The low correlation between the F1 scores of high-performance undergraduate and postgraduate classifiers and the corresponding ML classifiers suggest that the different test sets are not systemically easier or harder to classify.

We note that postgraduate classifiers outperformed undergraduates as a whole, although not significantly, reflecting their greater expertise. We also note that high-performance undergraduates (with and without feedback) outperformed postgraduates, although again not significantly. This suggests that selecting based on performance that is specific to the classification task can offset greater general expertise from more education.

### Performance by evaluation panel

**Fig. 4:**
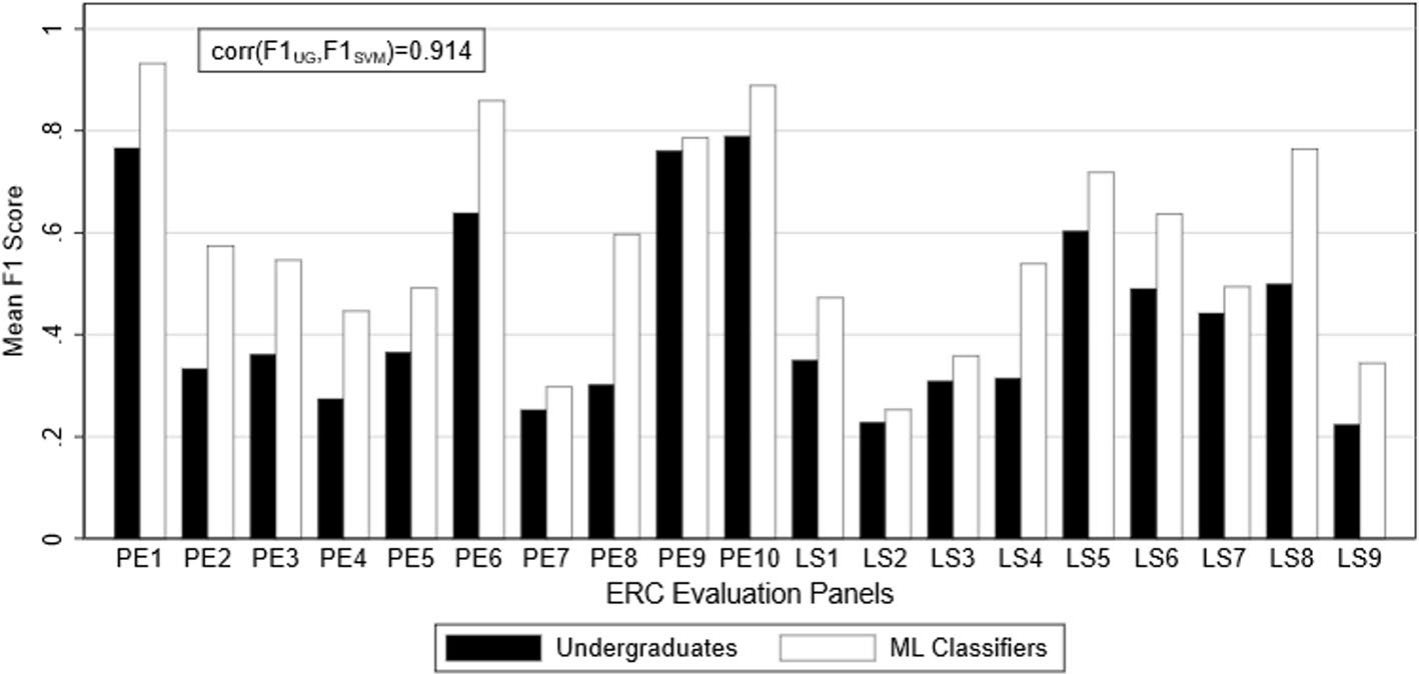
Comparison of undergraduate versus ML classification performance by panel

**Fig. 5:**
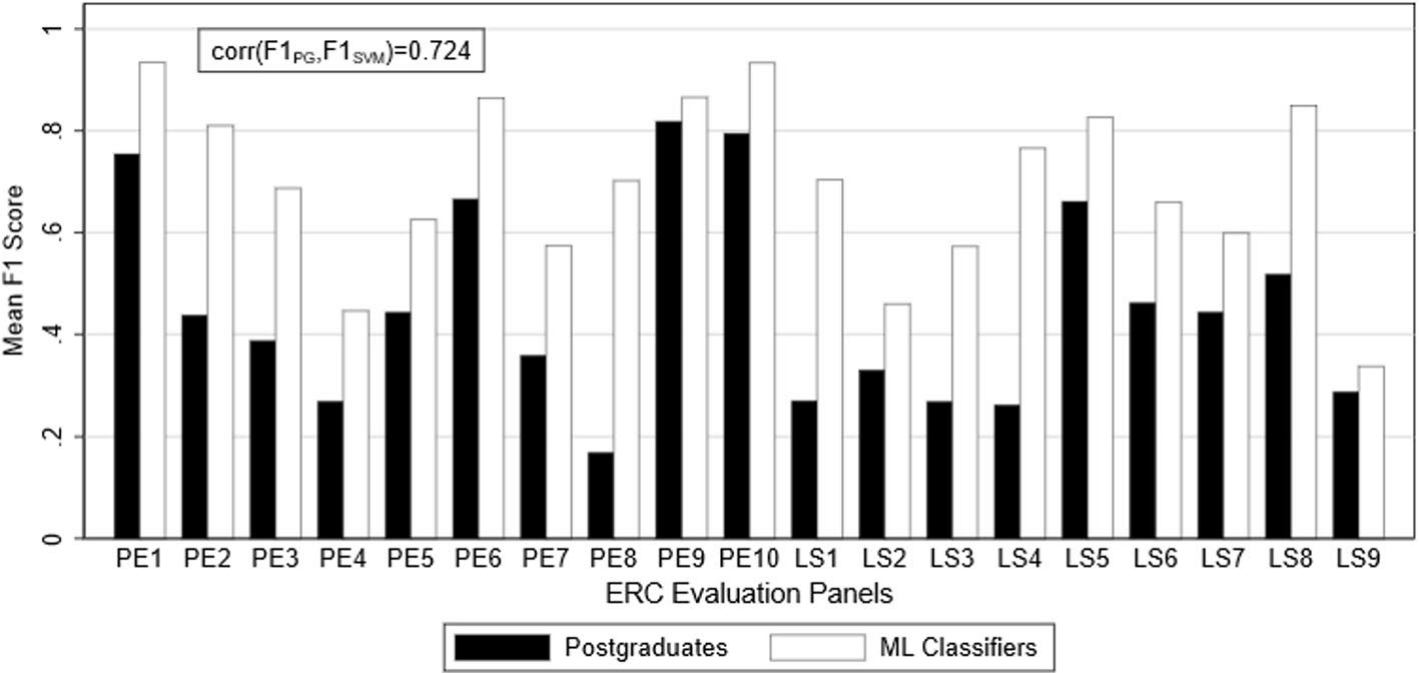
Comparison of postgraduate versus ML classification performance by panel

Figures 4 and 5 show the mean F1 scores of human and ML classifiers for each panel are shown in Fig. 4 for the undergraduates stage, and Fig. 5 for the postgraduates stage. ML classifiers perform consistently superior to human classifiers across all evaluation panels. There are some exceptions if the comparison is narrowed to selected human classifiers. For example, the high-performance undergraduates after feedback outperform ML classifiers in PE9 (Universe Sciences), with a mean F1 score of 0.86 compared to 0.78 for ML. This does not change the conclusion that ML classifiers broadly outperform, or at least do not underperform, human classifiers in each evaluation panel. The F1 scores of human and ML classifiers across evaluation panels are highly correlated in all stages (*ρ* = 0.914 in stage 1; *ρ* = 0.905 in stage 2; *ρ* = 0.931 in stage 3; *ρ* = 0.724 in stage 4). Evaluation panels that are difficult for ML classifiers to classify also give human classifiers problems.

### Inter-rater reliability

Table 3 shows Fleiss’ *κ* for ML and human classifiers in each stage and within each training set group for human classifiers. Fleiss’ *κ* is calculated over the subset of abstracts in common across all test sets. We drop any abstract that have missing classifications from any classifier as the standard error for Fleiss’ *κ* is only defined when all abstracts are classified by every classifier. A small number of abstracts that were mistakenly assigned to both the test set and training sets in the undergraduates stage are excluded.

**Table 3:** Fleiss’ *κ* of human versus ML classifiers

Stage	Trg. set	Human classifiers			ML classifiers		
		*κ*	SE	95% CI	*κ*	SE	95% CI
Undergraduates	All	0.363	0.000	[0.362, 0.364]	0.515	0.007	[0.501, 0.530]
	A	0.375	0.001	[0.372, 0.378]			
	B	0.373	0.002	[0.370, 0.376]			
	C	0.371	0.002	[0.368, 0.374]			
	D	0.376	0.002	[0.373, 0.379]			
High-performance undergraduates	All	0.395	0.001	[0.393, 0.398]	0.540	0.010	[0.521, 0.560]
	A	0.416	0.006	[0.405, 0.427]			
	B	0.398	0.005	[0.389, 0.407]			
	C	0.443	0.005	[0.432, 0.453]			
	D	0.361	0.005	[0.352, 0.370]			
High-performance undergraduates after feedback	All	0.391	0.001	[0.388, 0.393]	0.513	0.010	[0.493, 0.533]
	A	0.381	0.006	[0.370, 0.392]			
	B	0.377	0.005	[0.368, 0.387]			
	C	0.396	0.005	[0.385, 0.406]			
	D	0.407	0.005	[0.397, 0.416]			
Postgraduates	–	0.405	0.001	[0.402, 0.407]	0.913	0.001	[0.910, 0.915]

We observe that the Fleiss’ *κ* for ML classifiers are uniformly greater than that of human classifiers, and the confidence intervals for Fleiss’ *κ* for human and ML classifiers do not overlap. This suggests that ML classifiers are more reliable than human classifiers. The reliability of postgraduates is similar to the reliability of high-performance undergraduates, and the reliability of high-performance undergraduates is better than for all undergraduates. Further feedback does not seem to improve reliability. While the ML classifiers in the postgraduate stage have near-perfect reliability, this is expected because the ML classifier for each test set is trained using all left-out abstracts. This results in extensive overlaps in the training data across the ML classifiers, so we expect the learned parameters to also be similar, leading to similar predictions.

## Discussion and conclusion

ML classifiers are better at replicating the ground truth classification than human classifiers overall. Although Fig. 2 shows that there are individual human classifiers who perform as well as ML models trained on almost the entire corpus, selection and training through which such performance can be achieved require extensive resources. Only three human classifiers had F1 scores above 0.6 (see Figs. 2, 3), representing the top 5 percentile of performance among our human classifiers.

In contrast, Fig. 3 shows that ML classifiers, given sufficiently large training data sets, are consistently and highly accurate. ML performance is robust to variations in the training and test data. Furthermore, it is clear from Table 3 that human classifiers are not very reliable (*κ* mostly below 0.4) and are less reliable than ML classifiers.

Besides better classification accuracy and reliability, ML is also more efficient to train and use due to modern computing power. Each ML classifier in the Postgraduates stage took 1 h to train through multiprocessing on 24 CPU cores. This amount of computing power is easily available to most research teams today, and even ordinary personal computers are now capable of training ML models for many scientometric applications, albeit with more time required. After training, the ML classifiers classify 247 abstracts in less than 5 s. In contrast, in this study the fastest human classifiers took over 2 h to classify 247 abstracts in addition to a morning required for training.

Not only are supervised ML algorithms superior to humans in terms of time, accuracy and reliability for given data to train on and data to classify, but they also *scale* readily with *more* data. ML algorithms can be trained on larger datasets for greater accuracy, as was the case in our study, but more importantly, they can be applied to entire agency- or country-wide research corpora, even numbering in the hundreds of thousands of texts, to enable evaluation of funding agencies or even whole countries using a common classification, something that is simply infeasible with humans.

A limitation of our study is that it involves classification into a single panel or field. In principle, a research abstract could be classified in multiple fields and ML methods can be adapted to this, by assigning probability weights to multiple field classifications. However, in our case, the training dataset from the ERC Starting Grant only assigns research to a single main panel, as funding is disbursed strictly according to panels. Applying our methods using training datasets with multiple field classifications would be an interesting extension of our research.

## Appendix: Recruitment and characteristics of study participants

For the undergraduates stages, we recruited 63 undergraduate student assistants from Nanyang Technological University, a major research university in Singapore, for a full-day task. We sent out an email on our recruitment of undergraduates for our research abstract classification study to all undergraduates via the university’s mailing lists. The email included instructions to complete an attached example task requiring them to classify two research abstracts. This example task was to screen potential classifiers for aptitude. Just over one hundred undergraduates responded to the email and completed the attached example task. Of those, 63 ultimately agreed to participate by attending the full-day session. Undergraduates were paid S$120 (around 84 USD or 77 EUR) for the full-day session plus S$4 (2.80 USD or 2.50 EUR) per abstract correctly classified.

For the postgraduates stage, we recruited 26 Ph.D. students and postdoctoral researchers in STEM disciplines from Nanyang Technological University for a half-day task where they were given a test set to classify without any training or task exposure. We sent out an email on our recruitment of postgraduates for our research abstract classification study to all postgraduates in the College of Engineering through the assistance of the administrators in each School in the College.

Table 4 shows the distribution of participants by gender, subject area, and level of academic experience.

**Table 4:** Distribution of gender, subject area, and level of academic experience of participants

	All undergraduates	All postgraduates
*Gender*		
Male	34	18
Female	29	8
*Subject area*		
Health sciences	–	1
Life sciences	13	3
Physical sciences	46	22
Social sciences	4	–
*Year of study*		
1st	17	4
2nd	27	3
3rd	19	3
4th	–	5
5th	–	7
Postdoc	–	4
*N*	63	26

Table 5 shows the distribution of participants by detailed subject area according to the Scopus classification scheme.

**Table 5:** Distribution of detailed subject areas of participants

	All undergraduates	All postgraduates
*Scopus subject area*		
Biological sciences	4	–
Biochemistry	9	1
Business and management	3	–
Chemistry	1	–
Computer science	4	3
Earth sciences	–	1
Engineering	25	11
Environmental science	2	–
Material science	3	5
Mathematics	8	1
Medicine	–	1
Neuroscience	–	2
Physics	3	1
Social sciences	1	–
*N*	63	26
